# In Vivo Metabolite Profiling of a Purified Ellagitannin Isolated from *Polygonum capitatum* in Rats

**DOI:** 10.3390/molecules21091110

**Published:** 2016-08-24

**Authors:** Jing-Yi Ma, Xuelin Zhou, Jie Fu, Chi-Yu He, Ru Feng, Min Huang, Jia-Wen Shou, Zhen-Xiong Zhao, Xiao-Yang Li, Luye Zhang, Yang-Chao Chen, Yan Wang

**Affiliations:** 1 State Key Laboratory of Bioactive Substances and Functions of Natural Medicines, Institute of Materia Medica, Chinese Academy of Medical Sciences, Beijing 100050, China; majingy@gmail.com (J.-Y.M.); fujie@imm.ac.cn (J.F.); hechiyu@imm.ac.cn (C.-Y.H.); fengru@imm.ac.cn (R.F.); huangmin_0427@163.com (M.H.); shoujiawen@163.com (J.-W.S.); zhaozhenxiong@imm.ac.cn (Z.-X.Z.); lixiaoyang90@106.com (X.-Y.L.); zhanglvye1995@163.com (L.Z.); 2 School of Biomedical Sciences, Faculty of Medicine, The Chinese University of Hong Kong, Shatin, N.T., Hong Kong, China; peterxlzhou@gmail.com (X.Z.); yangchaochen@hotmail.com (Y.-C.C.)

**Keywords:** FR429, ellagitannin, metabolite profiling, LC/MS^n^-IT-TOF

## Abstract

Ellagitannin is a common compound in food and herbs, but there are few detailed studies on the metabolism of purified ellagitannins. FR429 is a purified ellagitannin with antitumor potential, which is from *Polygonum capitatum* Buch.-Ham.ex D. Don. The present study was designed to investigate the metabolic profiles of FR429 in rats in vivo. Using liquid chromatography coupled to ion trap time-of-flight mass spectrometry (LC/MS^n^-IT-TOF), total eight metabolites were found in rat bile and urine after intravenous administration of FR429, but could not be detected in plasma. These metabolites were ellagic acid, mono-methylated FR429, ellagic acid methyl ether glucuronide, ellagic acid methyl ether diglucuronide, ellagic acid dimethyl ether glucuronide, and ellagic acid dimethyl ether diglucuronide. It was concluded that methylation and subsequent glucuronidation were the major metabolic pathways of FR429 in rats in vivo. This is the first report on the in vivo metabolism of the purified ellagitannin in rats.

## 1. Introduction

Ellagitannins (ETs) are the hydrolyzable tannins enriched in fruits and herbs (especially berries and nuts). They are a family of bioactive polyphenols with large molecular weight, high polarity, and a core of glucose esterified with hexahydroxydiphenic acid [1,2]. They have multiple pharmacological effects such as antioxidant, antitumor, antiviral, antimicrobial, and immune-modulatory [3,4]. Previous studies mainly focused on the pharmacokinetics and metabolism of ETs after oral administration of ET-rich food or extracts since ETs are difficult to be purified. These studies have reported that the bioavailability of ETs is very low and rarely detected in plasma after normal consumption of ET-rich foods [5,6]. Only the metabolites, such as ellagic acid (EA), gallic acid (GA) and their metabolites, can be detected in vivo [7]. However, other ingredients in foods or extracts may affect the metabolic process of ETs when they were taken simultaneously, and the metabolism of purified ETs is not reported. Although it has been reported that ETs and their intermediate metabolites could be bio-transformed by gut microbiota [8,9], whether ETs can be metabolized in the liver has not been reported, and the pathways for ET metabolism after intravenous administration have not been elucidated completely.

The fresh plant of *Polygonum capitatum* Buch.-Ham.ex D. Don (*P. capitatum*; called “Tou-Hua-Liao” or “Si-Ji-Hong” in Chinese) growing in the southwest of China is a favorable vegetable food. Its dried plant is also widely used to treat various urologic disorders. FR429, a typical ET, is the most abundant component isolated from the ethanolic extracts of *P. capitatum* [10]. An in vitro study has found that FR429 was metabolized in rat liver cytosol and primary hepatocytes mainly through hydrolysis, methylation and sulfation [11]. In our previous study, FR429 dramatically inhibited tumor growth in hepatoma-xenografted mice after intraperitoneal administration (10 mg/kg) for two weeks. However, its in vitro IC_50_ value was very high (>100 μM) in hepatoma cells [12]. It is necessary to investigate the metabolic profiles of FR429 to determine whether the in vivo anti-cancer effects observed in xenografted mice are derived from its metabolites. The aim of our current study was to verify the in vitro metabolism and elimination pathways of FR429. A liquid chromatography-ion trap-time of flight mass spectrometry (LC/MS^n^-IT-TOF) method was used to analyze the metabolic profiles of FR429 in plasma, bile, and urine after its intravenous administration in rats in vivo.

## 2. Results and Discussion

### 2.1. Metabolites in Plasma

It has been reported that the metabolites such as ellagic acid were poorly detected in human plasma after consumed lots of ETs-rich fruit juice [7]. In this study, when compared to the control plasma, there were no metabolites found in plasma at different time points. This may be because the plasma concentrations of metabolites were much lower than the detection limit of LC/MS^n^-IT-TOF.

### 2.2. Metabolites in Bile

**Figure 1:**
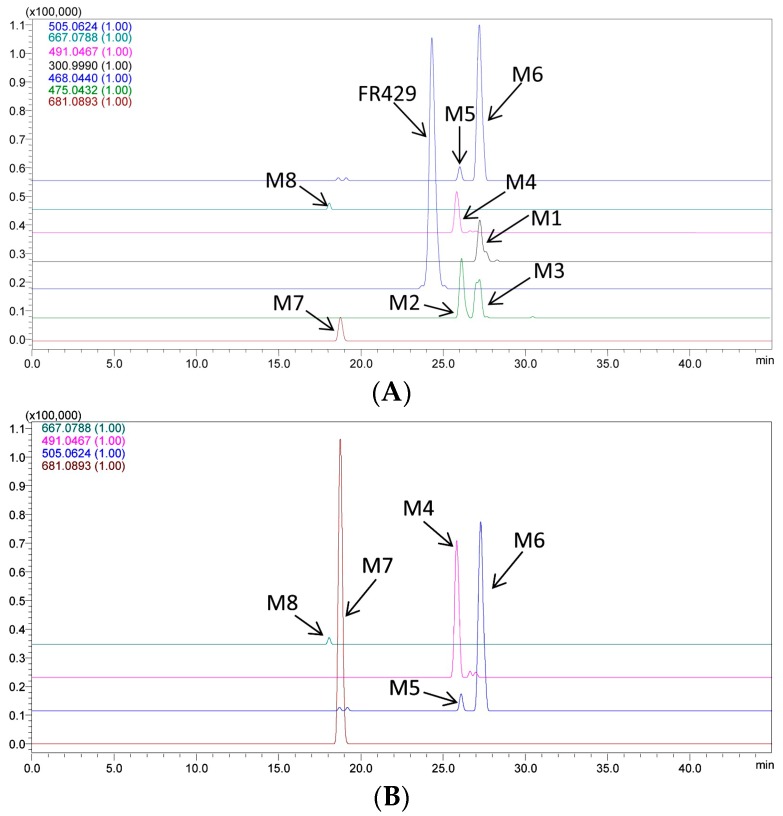
Representative extracted ion chromatograms (EICs) of the metabolites of FR429 in rats in vivo: (**A**) bile sample collected from 1 to 1.5 h; and (**B**) urine sample collected from 0 to 6 h.

The parent drug and eight metabolites were detected and characterized in rat bile samples after intravenous administration (Figure 1A). Two isomers of methyl ether FR429 and five Phase II conjugates of methylated EA were found (Table 1).

**Figure 2:**
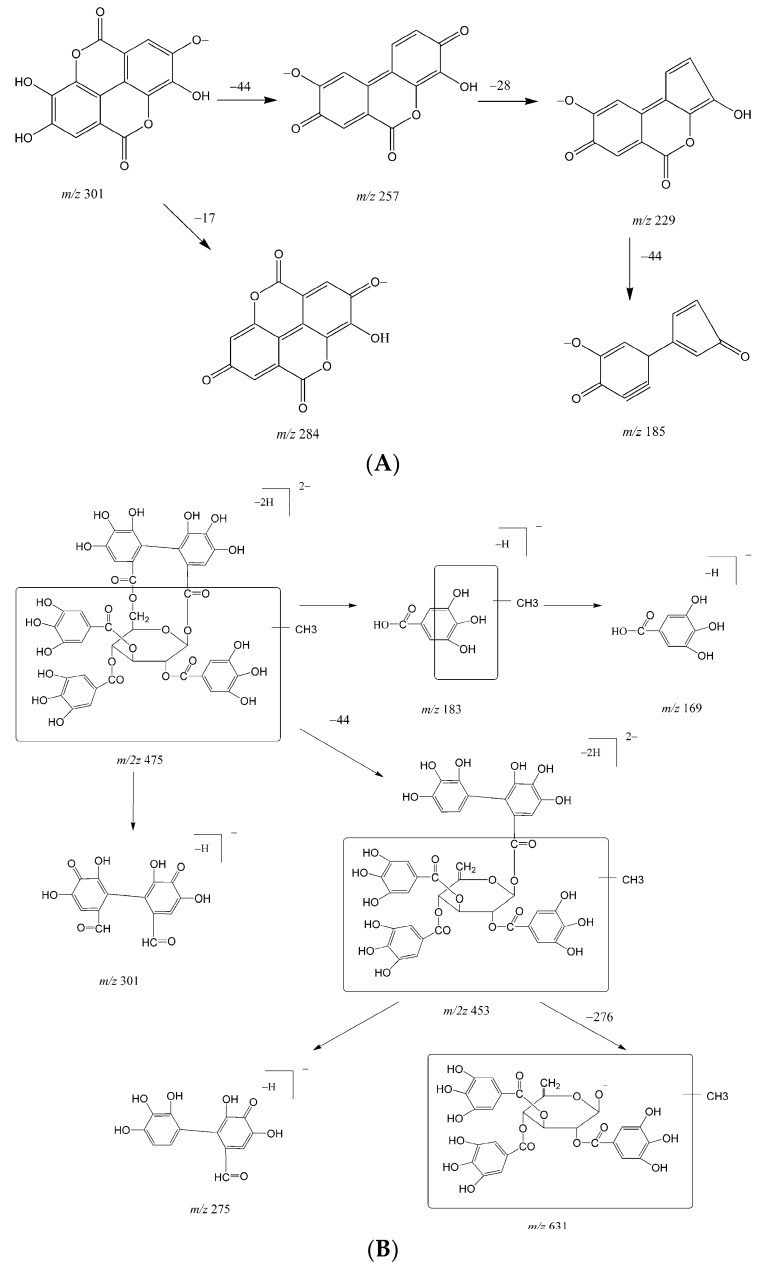

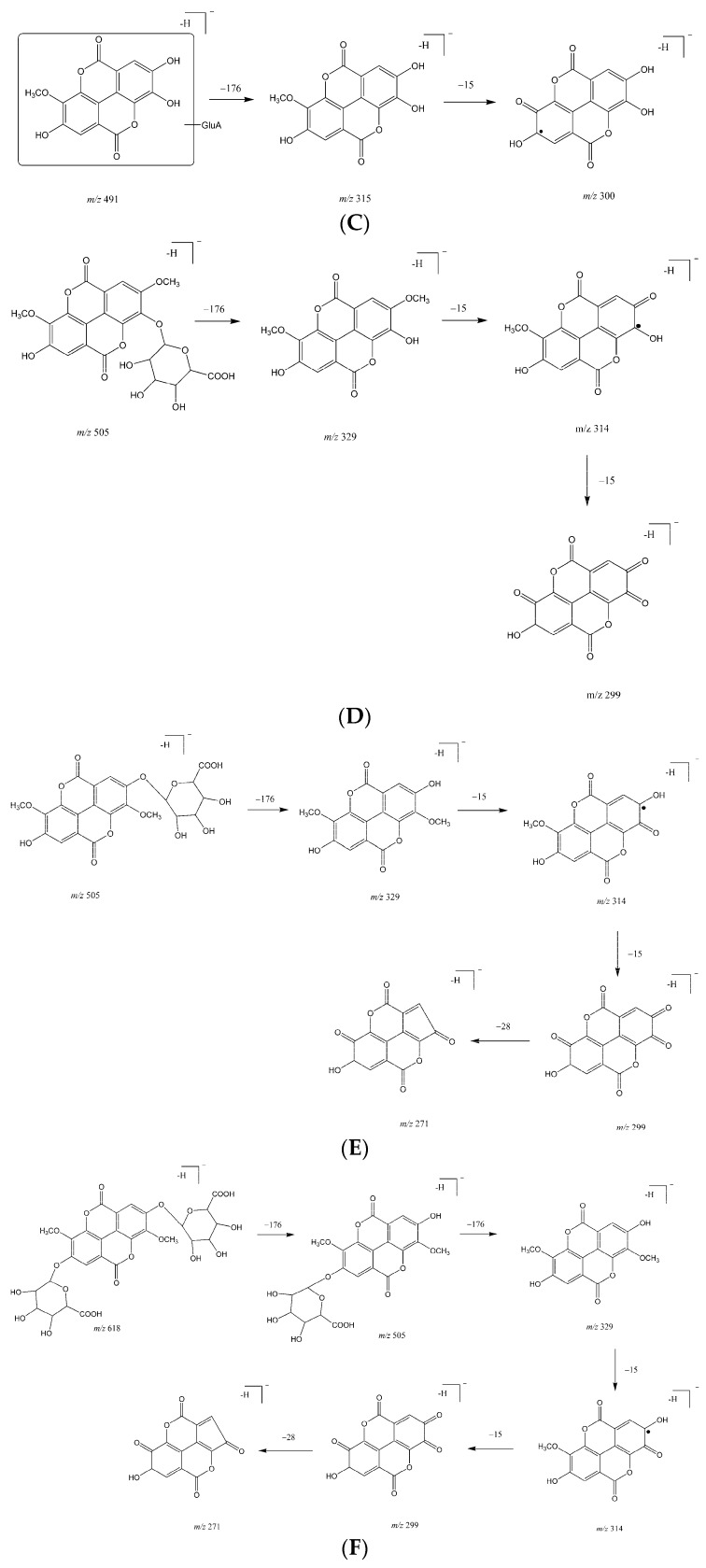

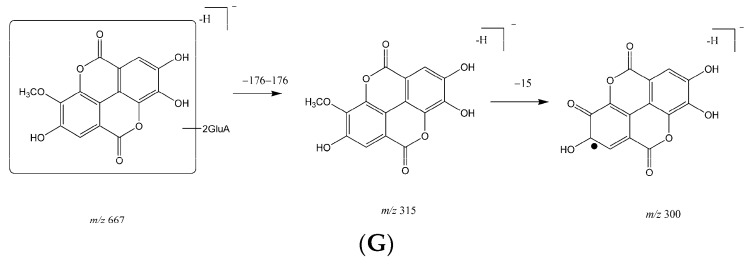
Fragmentation patterns of the in vivo metabolites of FR429: (**A**) M1; (**B**) M2 and M3; (**C**) M4; (**D**) M5; (**E**) M6; (**F**) M7; and (**G**) M8.

M1 was eluted at 27.0 min with [M − H]^−^ at *m*/*z* 300.9994. The fragments were *m*/*z* 284.00 ([M − H−OH]^−^), 257.01 ([M − H−CO_2_]^−^), 229.01 ([M − H−CO_2_−CO]^−^), and 185.03 ([M − H−2CO_2_−CO]^−^). The fragmentation pattern (Figure 2A) and retention time were the same as those of EA authentic standard, which confirms that M1 was ellagic acid.

M2 and M3 eluted at approximately 26.3 and 27.4 min, respectively. The [M − 2H]^2−^ for both M2 and M3 were observed at *m*/2*z* 475.05, which was 14 Da larger than [M − 2H]^2−^ for FR429. The fragmentation patterns of M2 and M3 were similar as previously reported (Figure 2B) [11]. Briefly, the fragment at *m*/*z* 183.03 indicates methylation of the galloyl group. The molecular ion fragmented to produce an ion at *m*/2*z* 453.06 by neutral loss of CO_2_, and further fragmented to *m*/*z* 631.08 by loss of HHDP group. Therefore, M2 and M3 were mono-methylated metabolites of FR429.

Metabolite M4 eluted at approximately 25.8 min with [M − H]^−^ at *m*/*z* 491.0469. The MS^2^ spectra of M4 showed a neutral loss of 176 Da, indicating that M4 was a glucuronide. The product ion at *m*/*z* 315.01 fragmented to *m*/*z* 299.99 by the loss of CH_3_ (15 Da) from the aglycone ions and eventually formed the fragments of EA. The fragmentation patterns are shown in Figure 2C. Therefore, M4 was identified as methyl-EA-glucuronide.

M5 and M6 eluted at 26.0 and 27.3 min, respectively. The [M − H]^−^ for both M5 and M6 were observed at *m*/*z* 505.06, which was 14 Da larger than that for M4. As shown in Figure 2D,E, neutral loss of 176 Da from [M − H]^−^ produced *m*/*z* 329.02, indicating that M5 and M6 were the glucuronic conjugates. The product ions at *m*/*z* 329.02, 314.01 were 14 Da larger than the corresponding product ions of M4, showing that methylation of a hydroxyl group had occurred on the phenyl moiety of M4. Therefore, M5 and M6 were identified as dimethyl-EA-glucuronide. 

As eluted at 18.4 min, [M − H]^–^ for M7 were observed at *m*/*z* 681.0947, and its fragmentation patterns are shown in Figure 2F. The MS^2^ spectra of M7 showed a successive neutral loss of 176 Da, indicating that M7 was di-glucuronic conjugates. The product ions including *m*/*z* 505, 329, 314, 299, and 270 were identical to M6, indicating that M7 was a glucuronide of M6. Therefore, M7 was identified as dimethyl-EA-diglucuronide.

M8 eluted at 17.8 min with a [M − H]^−^ ion at *m*/*z* 667.0827 which was 176 Da larger than M4, suggesting that M8 was a glucuronic conjugate. The fragments were consistent with those of M4; therefore, M8 was identified as methyl-EA-diglucuronide (Figure 2G).

### 2.3. Metabolites in Urine

At different time slots, the parent drug was not detected in urine. However, several glucuronides and diglucuronides of the methylated metabolites of EA were detected, including EA methyl ether glucuronide (M4), EA dimethyl ether glucuronide (M5 and M6), EA dimethyl ether diglucuronide (M7), and EA methyl ether diglucuronide (M8) (Figure 2B). The glucuronide conjugates of EA derivatives, as metabolites of FR429, are believed to be excreted through the kidneys into the urine.

**Figure 3:**
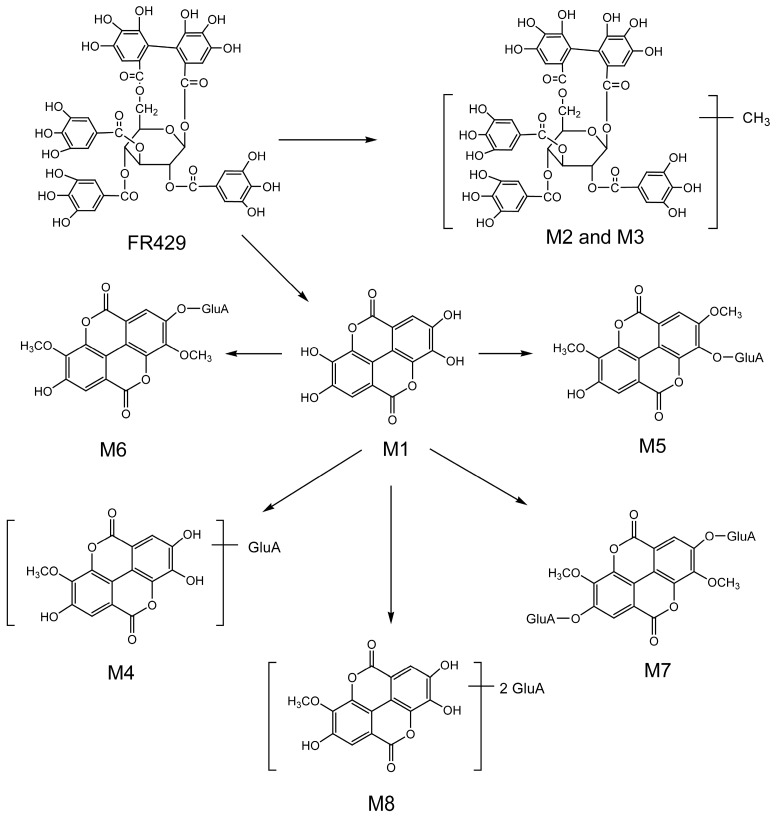
Proposed in vivo metabolic pathways of FR429 in rats.

As illustrated in the in vivo metabolic profiles of FR429 (Figure 3), eight metabolites were generated in bile during FR429 metabolism, of which EA may be an intermediate metabolite. Glucuronic conjugates of methylated EA were formed afterwards, including the major metabolites EA methyl ether glucuronide, EA dimethyl ether glucuronide, EA dimethyl ether diglucuronide, and traces of EA methyl ether diglucuronide. FR429 was excreted to bile in its original form as well as glucuronic conjugates of EA methylated metabolites. Traces of methylated FR429 were also observed in bile and their levels rapidly declined after administration. Small amounts of the glucuronic conjugates EA methyl ether glucuronide, EA dimethyl ether glucuronide, EA dimethyl ether diglucuronide, and EA methyl ether diglucuronide were excreted in urine. Thus, FR429 was transformed into more polar metabolites, which facilitates its elimination from the body. It seems that bile secretion is the main excretion pathway of FR429 and its metabolites, because more metabolites were found in bile, and small volume (50 μL) of bile sample was used for analysis when compared to those of plasma (200 μL) and urine (1.5 mL). In our previous in vitro study with liver cytosolic fraction, sulfated conjugates of FR429 have been found, but not in the current in vivo study. This suggested that glucuronidation was more preferable than sulfation as the Phase II metabolism pathway for FR429.

In addition, since EA and its metabolites, specifically including urolithins and dimethyl EA, have been shown to exhibit potent anti-cancer, antimicrobial, and antioxidant activities in vitro and in vivo [13,14,15,16], the in vivo anticancer property of FR429 was probably from its metabolites including EA and EA’s metabolites. Due to the poor solubility of EA, FR429 may be an alternative of EA for anti-cancer purpose as a pro-drug.

## 3. Experimental Section

### 3.1. Chemicals and Reagents

The authentic standard of ellagic acid (EA) was purchased from the National Institute for the Control of Pharmaceutical and Biological Products (Beijing, China). HPLC grade acetonitrile and formic acid were obtained from Labscan Analytical Science (Bangkok, Thailand), and ethyl acetate was from Fisher Chemicals (Leicester, UK). Distilled and deionized water was prepared using a Milli-Q purification system. All other unspecified chemicals were purchased from Sinopharm Chemical Reagent Co., Ltd. (Shanghai, China).

FR429 was prepared as described in our previous report [10]. Briefly, raw herb of *P. capitatum* was extracted by ultrasonic extraction with 70% ethanol, and then filtered. After the solvent was removed, the residue was re-suspended in water and then extracted by ethyl acetate. The residue was subjected to a Sephadex LH20 column (GE Healthcare Bio-Sciences AB, Uppsala, Sweden) with methanol to obtain FR429 fraction. This fraction was dried for pure FR429 (purity > 95%; HPLC-UV grade).

### 3.2. Animals

Male Sprague-Dawley rats (260–280 g, 7–8 weeks) were supplied by the Experimental Animal Science Department of Peking University Health Science Center (SCXK2006-0008, Beijing, China). Rats were given free access to food and water, and fasted overnight before experiments. The animals were maintained on a 12 h light/dark cycle (light on from 8:00 a.m. to 8:00 p.m.) at ambient temperature (22–24 °C) with 45% relative humidity. Before experiments and surgeries (except the urine collection experiment), all animals were generally anesthetized with urethane (20% *w*/*v* in normal saline; 6 mL/kg, i.p.). After general anesthesia for 1 h, carotid artery cannulation was performed for blood sampling as well as bile duct cannulation for bile sampling. All experimental procedures were approved by the Animal Experimentation Ethics Committee of Peking Union Medical College according to the guidelines for the Care and Use of Animals. 

### 3.3. LC/MS^n^-IT-TOF Analysis Conditions

Different from the triple-quadrupole tandem MS (tQ-MS) with high sensitivity but with low resolution and few information on fragments at MS^2^-MS^3^ stage, LC/MS^n^-IT-TOF is combined with ion trap and time of flight mass spectrometry, which means that it could provide MS^10^ stage at most with high resolution and precision (*m*/*z* has four decimal places) at each stage. Thus, LC/MS^n^-IT-TOF is better in qualitative analysis. The resolution of the instrument was larger than 10,000 at *m*/*z* 1000, and the resolution of precursor window for MS^n^ cycles was larger than 1000 at *m*/*z* 1000 with precursor isolation width 3 Da.

The identification analysis of FR429 and its metabolites were performed using an HPLC coupled to an ion trap time-of-flight mass spectrometer (LC/MS^n^-IT-TOF, Shimadzu Cooperation, Tokyo, Japan) with an Alltima C18 column (150 mm × 4.6 mm, 5 μm) as our previous study [11]. Briefly, the mobile phase consisted of acetonitrile (A) and 0.2% formic acid (B) (*v*/*v*) with a gradient elution: 0–10 min, 10% A; 30 min, 30% A; 40 min, 65% A; 45 min, 85% A; and 55 min, 85% A. The flow rate was 0.8 mL/min. For LC/MS^n^-IT-TOF analysis, an electrospray ionization (ESI) resource with a negative mode was used. The other parameters were set as follows: curved desolvation line (CDL) temperature, 200 °C; heat block temperature, 200 °C; detector voltage, 1.70 kV; nebulizing gas, 1.5 L/min; drying gas pressure, 110 kPa; and energy of collision-induced dissociation (CID), 50%. Mass spectra were obtained for MS^1^ in the range of *m*/*z* 100–1000. The MS^n^ data were collected in an automatic mode at three MS^n^ stages in the range of *m*/*z* 100–800 for MS^2^, 100–700 for MS^3^, and 100–500 for MS^4^, respectively.

### 3.4. Metabolites Identification in Plasma, Urine and Bile in Vivo

Rats were randomly separated into three groups for sample collection for plasma, bile, and urine study. FR429 (12 mg/kg; 10 mg/mL in 5% Tween-80 in water) was administrated via tail vein injection. Blood samples from six anesthetized rats (500 μL) were collected in a heparinized centrifuge tube via the carotid artery cannula at 0, 4, 10, 20, 40, 60, 90, 120, and 180 min. The blood samples were centrifuged at 3000 rpm for 10 min and stored immediately at −20 °C until analysis. Each plasma sample (200 μL) was intensely mixed with potassium dihydrogen phosphate (1 M; 30 μL), phosphoric acid (6 μL), and acetonitrile (204 μL), and then centrifuged at 14,000× *g* for 15 min. Then, the aliquot of the supernatant (25 μL) was subjected to analysis by LC/MS^n^-IT-TOF.

Using another five anesthetized rats, bile samples were collected via the bile duct as a control before drug administration. After injection as described above, bile samples were collected at 30 min intervals for 6 h. Bile samples (50 μL) were mixed with 90 μL of methanol-2% formic acid (9:1, *v*/*v*). The mixture was centrifuged at 10,000× *g* for 15 min, and the supernatant was filtered through a filter (0.45 μm). The sample (25 μL) was analyzed by LC/MS^n^-IT-TOF.

For urine sample collection, the third group of four rats (not anesthetized) were individually kept in the metabolic cages after tail injection. Urine samples (1.5 mL) were collected at 0, 6, 12, 24, and 48 h, respectively. All samples were added to the reverse phase C18 Sep-Pak cartridges (Waters, Milford, MA, USA) and subsequently washed with distilled water. Finally, metabolites were eluted with methanol. The methanolic fraction was dried under nitrogen flow, and the residue was dissolved in 300 μL of acetonitrile-0.2% formic acid (20:80) and then filtered through a filter (0.45 μm). The sample (25 μL) was analyzed by LC/MS^n^-IT-TOF.

## 4. Conclusions

In our previous in vitro study, methylated metabolites of FR429 were formed by cytosolic catechol-*O*-methyl transferase. In the present study, the glucuronic conjugates of these methylated metabolites were the major metabolites of FR429 in rat bile and urine in vivo after intravenous administration. It was concluded that methylation and subsequent glucuronidation were the major metabolic pathways of FR429 after intravenous administration. This is the first report to identify metabolites of mono-methylated ETs in vivo.

## Figures and Tables

**Table 1:** LC/MS^n^ data obtained for FR429 and its metabolites in vivo.

Code	*t*_R_ (min)	MS^1^ [M − H]^−^	Diff (ppm)	Fragments	Structure	Biological Matrix
FR429	24.5	468.0397(2)	7.99	370.0413(2), 275.0364, 300.9967, 169.0164	FR429	Plasma, bile
M1	27.0	300.9994	2.99	229.0147, 185.0256, 257.0099, 283.9998	ellagic acid	bile
M2	26.3	475.0526(2)	1.68	300.9961, 275.0175, 169.0181, 183.0300, 631.0838	FR429 methyl ether	bile
M3	27.4	475.0498(2)	4.21	300.9956, 275.0174, 169.0186, 453.0572(2), 631.0849	FR429 methyl ether	bile
M4	25.8	491.0469	0.41	315.0138, 299.9911	ellagic acid methyl ether glucuronide	bile, urine
M5	26.0	505.0661	7.33	329.0211, 314.0071, 298.9756	ellagic acid dimethyl ether glucuronide	bile, urine
M6	27.3	505.0635	2.18	329.0288, 314.0041, 298.9793, 270.9909	ellagic acid dimethyl ether glucuronide (isomer)	bile, urine
M7	18.4	681.0947	0.29	505.0564, 329.0250, 314.0029, 298.9759, 270.9918	ellagic acid dimethyl ether diglucuronide	bile, urine
M8	17.8	667.0827	5.85	315.0170, 299.9915	ellagic acid methyl ether diglucuronide	bile, urine

## References

[1] Ito H., Iguchi A., Hatano T. (2008). Identification of urinary and intestinal bacterial metabolites of ellagitannin Geraniin in rats. J. Agric. Food Chem..

[2] Bakkalbasi E., Mentes O., Artik N. (2009). Food ellagitannins-occurrence, effects of processing and storage. Crit. Rev. Food Sci. Nutr..

[3] Okabe S., Suganuma M., Imayoshi Y., Taniguchi S., Yoshida T., Fujiki H. (2001). New TNF-alpha releasing inhibitors, geraniin and corilagin, in leaves of Acer nikoense, Megusurino-ki. Biol. Pharm. Bull..

[4] Nakashima H., Murakami T., Yamamoto N., Sakagami H., Tanuma S., Hatano T., Yoshida T., Okuda T. (1992). Inhibition of Human Immunodeficiency Viral Replication by Tannins and Related-Compounds. Antivir. Res..

[5] Boukharta M., Jalbert G., Castonguay A. (1992). Biodistribution of ellagic acid and dose-related inhibition of lung tumorigenesis in A/J mice. Nutr. Cancer.

[6] Cerda B., Llorach R., Ceron J.J., Espin J.C., Tomas-Barberan F.A. (2003). Evaluation of the bioavailability and metabolism in the rat of punicalagin, an antioxidant polyphenol from pomegranate juice. Eur. J. Nutr..

[7] Seeram N.P., Zhang Y., McKeever R., Henning S.M., Lee R.P., Suchard M.A., Li Z., Chen S., Thames G., Zerlin A. (2008). Pomegranate juice and extracts provide similar levels of plasma and urinary ellagitannin metabolites in human subjects. J. Med. Food.

[8] Garcia-Munoz C., Vaillant F. (2014). Metabolic fate of ellagitannins: Implications for health, and research perspectives for innovative functional foods. Crit. Rev. Food Sci. Nutr..

[9] Fu J., Ma J.Y., Zhang X.F., Wang Y., Feng R., Chen Y.C., Tan X.S., Zhang Y.Y., Sun Y.P., Zhou Y. (2012). Identification of metabolites of FR429, a potential antitumor ellagitannin, transformed by rat intestinal bacteria in vitro, based on liquid chromatography-ion trap-time of flight mass spectrometry analysis. J. Pharm. Biomed. Anal..

[10] Yang B., Feng R., Wang W., Zhang L., Ye X., Wang Y., Wang M. (2008). Quantitative analysis of three active constituents in Miao regional herb, *Polygonum capitatum* by HPLC/DAD/MS. Chin. J. Pharm. Anal..

[11] Ma J.Y., Zhou X., Fu J., Hu T., Or P.M.Y., Feng R., He C.Y., Chen W.J., Zhang X., Chen Y. (2014). Metabolite profiling analysis of FR429, an ellagitannin purified from *Polygonum capitatum*, in rat and human liver microsomes, cytosol and rat primary hepatocytes in vitro. Chem. Biol. Interact..

[12] Wang Y., Ma J., Chow S.C., Li C.H., Xiao Z., Feng R., Fu J., Chen Y. (2014). A potential antitumor ellagitannin, davidiin, inhibited hepatocellular tumor growth by targeting EZH2. Tumour Biol..

[13] Wang N., Wang Z.Y., Mo S.L., Loo T.Y., Wang D.M., Luo H.B., Yang D.P., Chen Y.L., Shen J.G., Chen J.P. (2012). Ellagic acid, a phenolic compound, exerts anti-angiogenesis effects via VEGFR-2 signaling pathway in breast cancer. Breast Cancer Res. Treat..

[14] Bialonska D., Kasimsetty S.G., Khan S.I., Ferreira D. (2009). Urolithins, intestinal microbial metabolites of Pomegranate ellagitannins, exhibit potent antioxidant activity in a cell-based assay. J. Agric. Food Chem..

[15] Gonzalez-Sarrias A., Gimenez-Bastida J.A., Garcia-Conesa M.T., Gomez-Sanchez M.B., Garcia-Talavera N.V., Gil-Izquierdo A., Sanchez-Alvarez C., Fontana-Compiano L.O., Morga-Egea J.P., Pastor-Quirante F.A. (2010). Occurrence of urolithins, gut microbiota ellagic acid metabolites and proliferation markers expression response in the human prostate gland upon consumption of walnuts and pomegranate juice. Mol. Nutr. Food Res..

[16] Rahman A., Ngounou F.N., Choudhary M.I., Malik S., Makhmoor T., Nur E.A.M., Zareen S., Lontsi D., Ayafor J.F., Sondengam B.L. (2001). New antioxidant and antimicrobial ellagic acid derivatives from Pteleopsis hylodendron. Planta Med..

